# Fetal XCMR: a numerical phantom for fetal cardiovascular magnetic resonance imaging

**DOI:** 10.1186/s12968-019-0539-2

**Published:** 2019-05-23

**Authors:** Christopher W. Roy, Davide Marini, William Paul Segars, Mike Seed, Christopher K. Macgowan

**Affiliations:** 1 Department of Radiology, Lausanne University Hospital (CHUV) and University of Lausanne (UNIL), Lausanne, Vaud Switzerland; 2 Department of Medical Biophysics, University of Toronto, Toronto, Ontario Canada; 3 Division of Translational Medicine, Peter Gilgan Centre for Research & Learning, The Hospital for Sick Children, 686 Bay Street, Toronto, ON M5G 0A4 Canada; 4 Division of Pediatric Cardiology, The Hospital for Sick Children, Toronto, Ontario Canada; 5 Department of Radiology, Duke University Medical Center, Durham, North Carolina USA; 6 Departments of Pediatrics and Diagnostic Imaging, University of Toronto, Toronto, Ontario Canada

**Keywords:** Fetal cardiovascular magnetic resonance imaging, Numerical simulation, Physiological motion, Golden angle radial, Motion correction, Post-processing

## Abstract

**Background:**

Validating new techniques for fetal cardiovascular magnetic resonance (CMR) is challenging due to random fetal movement that precludes repeat measurements. Consequently, fetal CMR development has been largely performed using physical phantoms or postnatal volunteers. In this work, we present an open-source simulation designed to aid in the development and validation of new approaches for fetal CMR. Our approach, fetal extended Cardiac-Torso cardiovascular magnetic resonance imaging (Fetal XCMR), builds on established methods for simulating CMR acquisitions but is tailored toward the dynamic physiology of the fetal heart and body. We present comparisons between the Fetal XCMR phantom and data acquired in utero, resulting in image quality, anatomy, tissue signals and contrast.

**Methods:**

Existing extended Cardiac-Torso models are modified to create maternal and fetal anatomy, combined according to simulated motion, mapped to CMR contrast, and converted to CMR data. To provide a comparison between the proposed simulation and experimental fetal CMR images acquired in utero, images from a typical scan of a pregnant woman are included and simulated acquisitions were generated using matching CMR parameters, motion and noise levels. Three reconstruction (static, real-time, and CINE), and two motion estimation methods (translational motion, fetal heart rate) from data acquired in transverse, sagittal, coronal, and short-axis planes of the fetal heart were performed to compare to in utero acquisitions and demonstrate feasibility of the proposed simulation framework.

**Results:**

Overall, CMR contrast, morphologies, and relative proportions of the maternal and fetal anatomy are well represented by the Fetal XCMR images when comparing the simulation to static images acquired in utero. Additionally, visualization of maternal respiratory and fetal cardiac motion is comparable between Fetal XCMR and in utero real-time images. Finally, high quality CINE image reconstructions provide excellent delineation of fetal cardiac anatomy and temporal dynamics for both data types.

**Conclusion:**

The fetal CMR phantom provides a new method for evaluating fetal CMR acquisition and reconstruction methods by simulating the underlying anatomy and physiology. As the field of fetal CMR continues to grow, new methods will become available and require careful validation. The fetal CMR phantom is therefore a powerful and convenient tool in the continued development of fetal cardiac imaging.

**Electronic supplementary material:**

The online version of this article (10.1186/s12968-019-0539-2) contains supplementary material, which is available to authorized users.

## Background

Assessing the human fetal heart with cardiovascular magnetic resonance (CMR) requires high-resolution acquisitions and reconstructions that are robust to artifacts from maternal respiration and gross fetal movement. Additionally, a fetal electrocardiogram signal for cardiac gating is not readily available in the CMR environment, requiring alternative strategies for conventional CINE imaging of the fetal heart. As a result, a growing number of studies have proposed methods for accelerated imaging, motion compensation, and image-based gating strategies to enable diagnostically useful fetal CMR images [1–7]. Still, validating new fetal CMR techniques is challenging, as stochastic fetal motion precludes repeat measurements, making it difficult to evaluate the parameter space for a given acquisition or reconstruction routine. Consequently, fetal CMR development has been largely performed using physical phantoms or postnatal healthy subjects, resulting in a lack of widely available fetal-specific reference models and minimal inter-study validation.

Numerical phantoms, including analytical and voxel-based models have been widely used to validate advanced acquisition and image reconstruction strategies in the broader CMR community [8–13]. Analytical phantoms, based on the continuous Fourier transform, provide an accurate depiction of k-space acquisitions. However, analytical phantoms are generally confined to simplistic shapes and rarely incorporate motion. Conversely, voxel-based phantoms provide more realistic simulations of dynamic anatomy but are limited by the discrete Fourier transform, and are constrained to the resolution and acquisition parameters of the images from which the phantom is derived.

Recently, a combined analytical and voxel-based approach for simulating CMR acquisitions called MRXCAT has been used to validate a variety of CMR strategies [14]. MRXCAT simulates a CMR acquisition based on user-supplied scan parameters, where the anatomy is defined by the extended Cardiac-Torso (XCAT) phantom, a high-resolution depiction of anatomical objects derived from the Visible Human Project of the National Library of Medicine [12, 15]. The MRXCAT phantom maps the realistic anatomical regions simulated by XCAT to CMR images using known CMR relaxation times for a variety of tissues. Additionally, it can generate multiple time points representing variable respiratory and cardiac motion. Still, the size of the fetal cardiac anatomy, relatively high fetal heart rate, range of motion, and large field of view required to capture the maternal abdomen, is not currently represented by the MRXCAT phantom, Furthermore, while numerical models of pregnant women have been used to assess radiofrequency exposure and temperature increases in CMR, a framework for fetal CMR image reconstruction does not currently exist [16, 17].

In this work, we present an open-source numerical phantom designed to aid in the development and validation of new approaches for fetal CMR. Our phantom referred hereafter as the fetal extended Cardiac-Torso cardiovascular magnetic resonance imaging (Fetal XCMR) phantom, combines two independent four-dimensional (4D, x, y, z, t) XCAT models of human anatomy (maternal and fetal) with a flexible simulation of two-dimensional (2D) multi-slice Cartesian and radial CMR acquisitions. It can be downloaded from: https://github.com/cwroy/Fetal-XCMR/.

Variable physiological parameters are included to control the level of maternal respiration and fetal movement. We present comparisons between the Fetal XCMR phantom and fetal CMR data acquired in utero. Additionally, reconstructions of undersampled acquisitions (compressed sensing) with imaged-based motion (translational registration) and gating estimates (metric optimized gating) are presented to highlight potential applications of the phantom [3].

## Methods

**Fig. 1:**
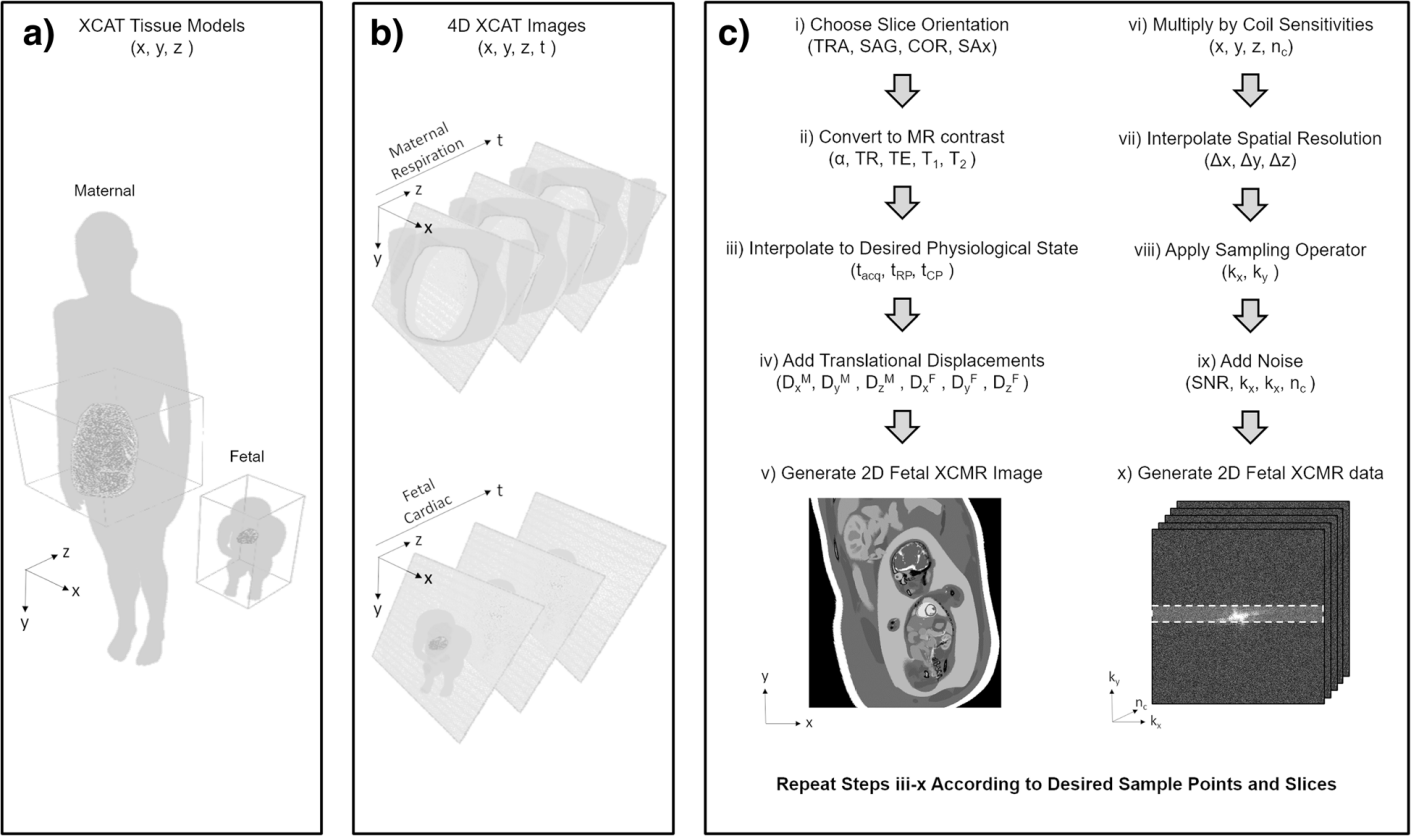
Workflow for creating fetal CMR phantom data. **a** High resolution numerical phantoms of maternal and fetal anatomy are derived from a female XCAT model modified to include an extended abdomen and amniotic fluid (maternal), and an infant XCAT model with modified limbs. **b** Using the models from (**a**), independent 4D images are generated that cover the maternal abdomen and full fetal anatomy over a complete maternal respiratory cycle and fetal cardiac cycle. **c** For each time point in the simulated acquisition, a Fetal XCMR image with CMR contrast and physiological motion is defined by user-selected acquisition parameters and motion amplitudes respectively. Fetal XCMR k-space data is then generated from the previously defined image and time point and steps iii-x are repeated to create a complete Fetal XCMR data set. All steps from (**c**) contain user modifiable parameters as listed in Table 1

Figure 1 provides an overview of the proposed workflow for simulating Fetal XCMR acquisitions, organized into four stages. First, existing XCAT models are modified to create maternal and approximate fetal anatomy (Fig. 1a). Second, 4D image arrays are generated from the modified XCAT models to form the basis of the Fetal XCMR phantom (Fig. 1b). Third, independent 4D XCAT arrays are combined and XCAT tissue values are mapped to CMR contrast (Fig. 1c). Fourth, CMR data is calculated from the image in the previous stage (Fig. 1d). In principle, stages one and two are performed once to generate the numerical models for a given base resolution, while stages three and four are repeated to generate simulated k-space according to a user-selected sampling trajectory and reordering scheme. The following describes, in greater detail, the individual steps of the proposed simulation workflow.

High resolution numerical phantoms of normal human maternal and fetal anatomy were created by modifying XCAT tissue models of adult female and infant male anatomy respectively [12]. The maternal tissue model includes an extended abdomen and amniotic fluid while the fetal model features fluid filled lungs, flexed limbs and approximate fetal lie (Fig. 1a). Additional features such as the placenta, fetal-specific blood vessels and shunts, and morphology of the right ventricle, were not included in the current implantation but may be of interest in cases where complex pathologies need to be simulated. As fetal-specific XCAT tissue models become available, their incorporation into the Fetal XCMR phantom will be straightforward due to the modular nature of the proposed framework. Nevertheless, the current model was designed to develop and validate acquisition and reconstruction methods rather than evaluate specific cardiac abnormalities.

Using the maternal and Fetal XCAT tissue models, two independent 4D XCAT image arrays are generated with spatial coverage of the maternal abdomen and full fetal anatomy, and temporal coverage of a representative maternal respiratory cycle and fetal cardiac cycle (Fig. 1b).

To simulate an acquisition with a user-selected slice orientation, the 4D arrays are rotated accordingly (Fig. 1c. i). XCAT tissue values are then converted to CMR contrast using the following equation for CMR signal (S) with a balanced steady-state free precession (bSSFP) sequence:

$$ \mathrm{S}=\frac{\sin\ \alpha \left(1-{\mathrm{e}}^{-\frac{\mathrm{T}\mathrm{R}}{\mathrm{T}1}}\right){\mathrm{e}}^{-\frac{\mathrm{T}\mathrm{E}}{\mathrm{T}2}}}{1-\left({\mathrm{e}}^{-\frac{\mathrm{T}\mathrm{R}}{\mathrm{T}1}}-{\mathrm{e}}^{-\frac{\mathrm{T}\mathrm{R}}{\mathrm{T}2}}\right)\left(\cos\ \alpha -1\right)} $$

where α, repetition time (TR), and echo time (TE) are the user-selected flip angle, repetition time, and echo time respectively, and T1 and T2 are the relaxation times which are derived from literature values for known adult and fetal tissues at 1.5 Tesla (Fig. 1c. ii) [18, 19]. For a given time point in the simulated acquisition (t_acq_), the maternal and fetal 4D arrays, now with CMR contrast, are interpolated to a single maternal respiratory phase (t_RP_) and fetal cardiac phase (t_CP_) (Fig. 1c. iii). Maternal respiratory phases are calculated using a constant respiratory rate that is randomly generated from a physiologically normal range (12–20 cycles-per-minute), while fetal cardiac phases are also derived from a normal range (110–180 bpm) but include a model of heart rate variation based on a bounded random walk [20]. Following interpolation of the 4D arrays to single physiological phases, the 3D position of the simulated fetal anatomy is translated according to the magnitude of maternal respiration (D_x_^M^, D_y_^M^, D_z_^M^) and a model of gross fetal movement (D_x_^F^, D_y_^F^, D_z_^F^) which is also based on a bounded random walk (Fig. 1c. iv). For both sources of motion, user-selected amplitudes dictate the level of displacement in each direction. The shifted fetal array is now added to the maternal array creating a 2D in utero image of the fetal cardiac anatomy CMR contrast (Fig. 1c. v).

To simulate the CMR acquisition at time t_acq_, the previously described 2D image is multiplied by simulated coil sensitives (Fig. 1d. i), the image resolution is modified according to user-selected values (Fig. 1d. ii), a sampling operator (Cartesian: Fourier transform, radial: non-uniform Fourier transform [21]) derives k-space from the now multi-coil image (Fig. 1d iii), and finally Gaussian noise is added (Fig. 1d iv) creating realistic multi-coil fetal CMR data (Fig. 1d v). The steps outlined in Fig. 1c, d are repeated according to the desired number of phase-encoding lines and time frames (Cartesian) or spokes (radial), as well as other user-selected acquisition parameters that would be found on a typical CMR scanner to simulate a complete fetal CMR dataset. Table 1 provides a complete overview of the user-selected parameters available in the Fetal XCMR framework including those explained by Fig. 1. The user-selected respiratory and gross fetal movement amplitudes provide flexible control of the motion level during the acquisition.

**Table 1:** Overview of user inputs for the Fetal XCMR phantom that define the orientation of the simulated data, the CMR acquisition, and the underlying motion models

Slice Orientation	
Transverse, Sagittal, Coronal, Short-axis	
Acquisition Parameters	
Sampling (Cartesian, Radial)	slice thickness (Δz)
Flip angle (α)	number of coils
Repetition time (TR)	number of slices
Echo time (TE)	number of spokes
In-plane resolution (Δx, Δy)	number of frames
Physiological Parameters	
Respiration motion amplitude (D_x_^M^, D_y_^M^, D_z_^M^)	fetal movement amplitude (D_x_^F^, D_y_^F^, D_z_^F^)

### Comparison of fetal XCMR and in utero acquisitions

To provide a comparison between the proposed simulation and experimental fetal CMR images acquired in utero, images from a typical scan of a pregnant woman (35 weeks gestational age) are included. This subject participated in a larger institutional Research Ethics Board approved study with written consent. Simulated acquisitions were generated using matching CMR parameters, slice prescriptions, and simulated motion levels which were chosen to reflect those observed in utero.

A multi-slice 2D bSSFP sequence with continuous golden angle radial sampling was used for the in-utero scans and replicated by the Fetal XCMR framework. This trajectory was chosen due to its flexible reconstruction options, as described below, allowing for a comprehensive comparison between the Fetal XCMR phantom and in utero acquisitions. Experimental slices were prescribed in transverse, sagittal, coronal, and short-axis planes of the fetal heart on a 1.5 T clinical CMR system using both body and spine matrices for reception with approximately thirty active channels (Avanto Fit, Siemens Healthineers, Erlangen, Germany). For each plane, a stack of 10–20 slices was acquired spanning the heart. All scans were acquired free-breathing with the following CMR parameters: flip angle: 70°, acquired spokes: 1500, TR/TE: 4.95/2.41 ms, samples per spoke: 256 (with two-fold oversampling to avoid wrap), field-of-view: 256 × 256 mm^2^, spatial resolution: 1 × 1 × 4 mm^3^, acquisition length per slice: 7 s.

### CMR reconstruction

For both simulated and in utero acquisitions, three reconstructions were performed in a manner described previously for golden angle radial fetal CMR data [3]. Briefly, static 2D images were reconstructed using all acquired spokes, 2D real-time images were reconstructed using a sequential sliding windows of spokes (15 spokes per frame with 10 spokes of view sharing), and finally CINE images of a representative heart beat were reconstructed (~ 75 spokes per frame depending on heart rate with no view sharing). For real-time and CINE image reconstructions, compressed sensing was used to reduce streaking artifacts using the algorithm and regularization weights described previously [22, 23]. Additionally, motion compensation and retrospective cardiac gating were used in the CINE reconstructions by extracting motion estimations and the fetal heart rate from the real-time images [1, 3].

### Fetal XCMR computation

The Fetal XCMR framework was implemented in MATLAB (MathWorks, Natick, Massachusetts, USA) on a personal computer with a single i7–6700 processor (Intel Corporation, Santa Clara, California, USA; clock speed 2.60 GHz; 4 cores) and 32 GB of RAM. Both maternal and fetal XCAT anatomies were generate for 50 time-points to create representative respiratory and cardiac cycles respectively. The spatial resolution for both anatomies was 1 mm isotropic. Note that once these XCAT tissue models were generated, all subsequent acquisitions could be simulated from these stored 4D XCAT images. Radial k-space was generated using the non-uniform fast Fourier transform (NUFFT) implementation by J. Fessler which is available online [21]. The computational times for both XCAT tissue model and XCMR phantom generation were recorded.

## Results

Generation of maternal and fetal 4D XCAT images as described by Fig. 1b took approximately 80 and 30 min respectively. For the comparison to in utero data, each Fetal XCMR acquisition took approximately 10–15 min to generate depending on the orientation, and half of that computational time was spent converting from Fetal XCMR images to radial k-space using the non-uniform fast Fourier transform.

**Fig. 2:**
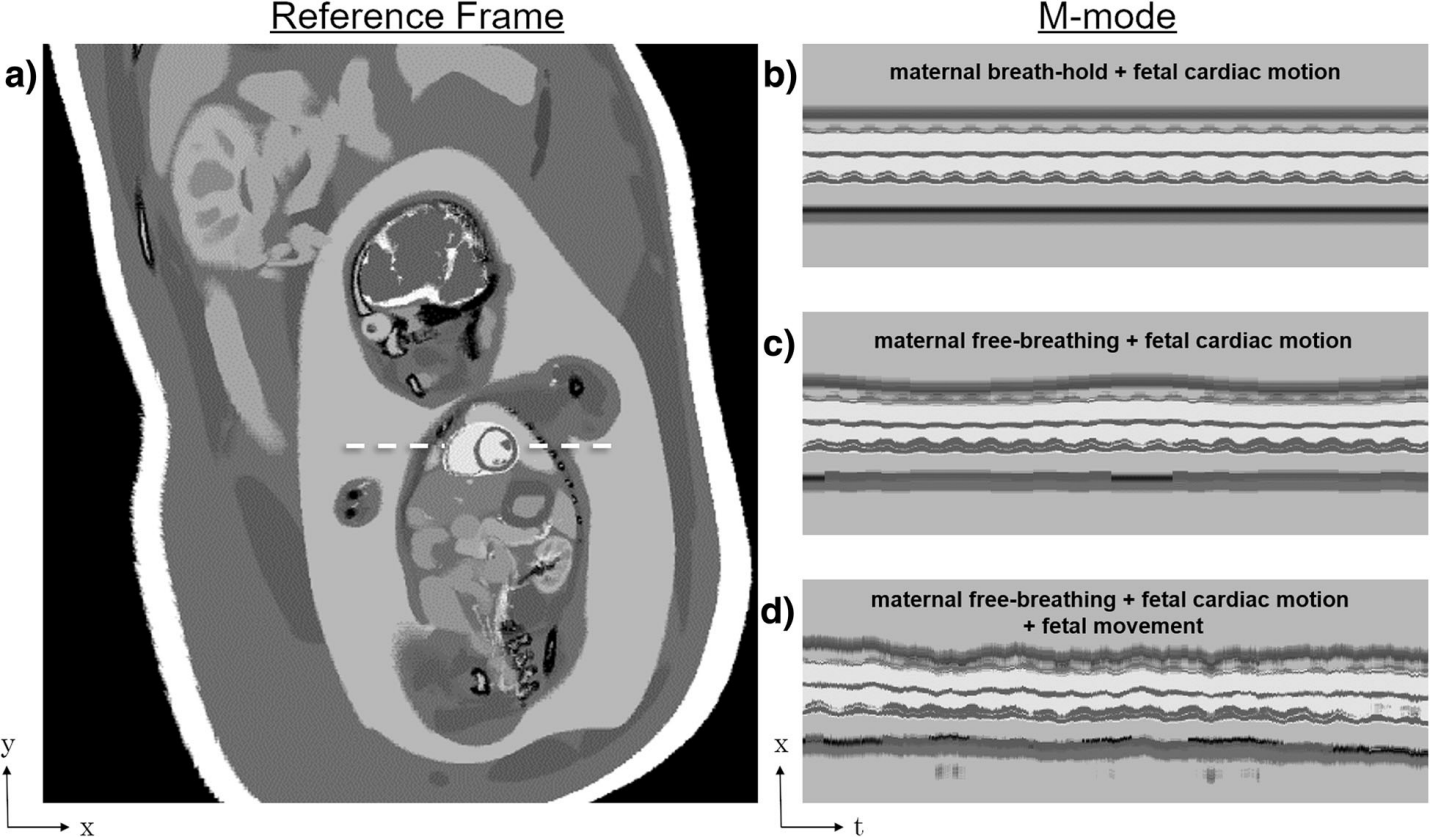
Example motion states using the Fetal XCMR phantom. Fetal XCMR images in a short-axis orientation (**a**) were generated over multiple timepoints to illustrate the temporal dynamics (M-mode) along the dashed-line in (**a**) for a simulated maternal breath-hold (**b**) demonstrating only fetal cardiac motion, a simulated free-breathing acquisition (**c**) demonstrating both maternal respiration and fetal cardiac motion, and a free-breathing acquisition with gross fetal movement (**d**) demonstrating three independent motions


Figure 2 illustrates three typical user-controlled motion states: maternal breath-hold (Fig. 2b), maternal free-breathing (Fig. 2c), and maternal free-breathing with gross fetal movement (Fig. 2d). Movies corresponding to these exemplary reconstructions are available as Additional file 1: Video S1.

Additional file 1:**Video S1.** Demonstration of three motion states that can be simulated from user-selected respiratory and gross fetal movement amplitudes providing flexible control of the motion level during the acquisition. a) Maternal breath-hold, b) maternal free-breathing, and c) maternal free-breathing with gross fetal movement. (MP4 11400 kb)


**Fig. 3:**
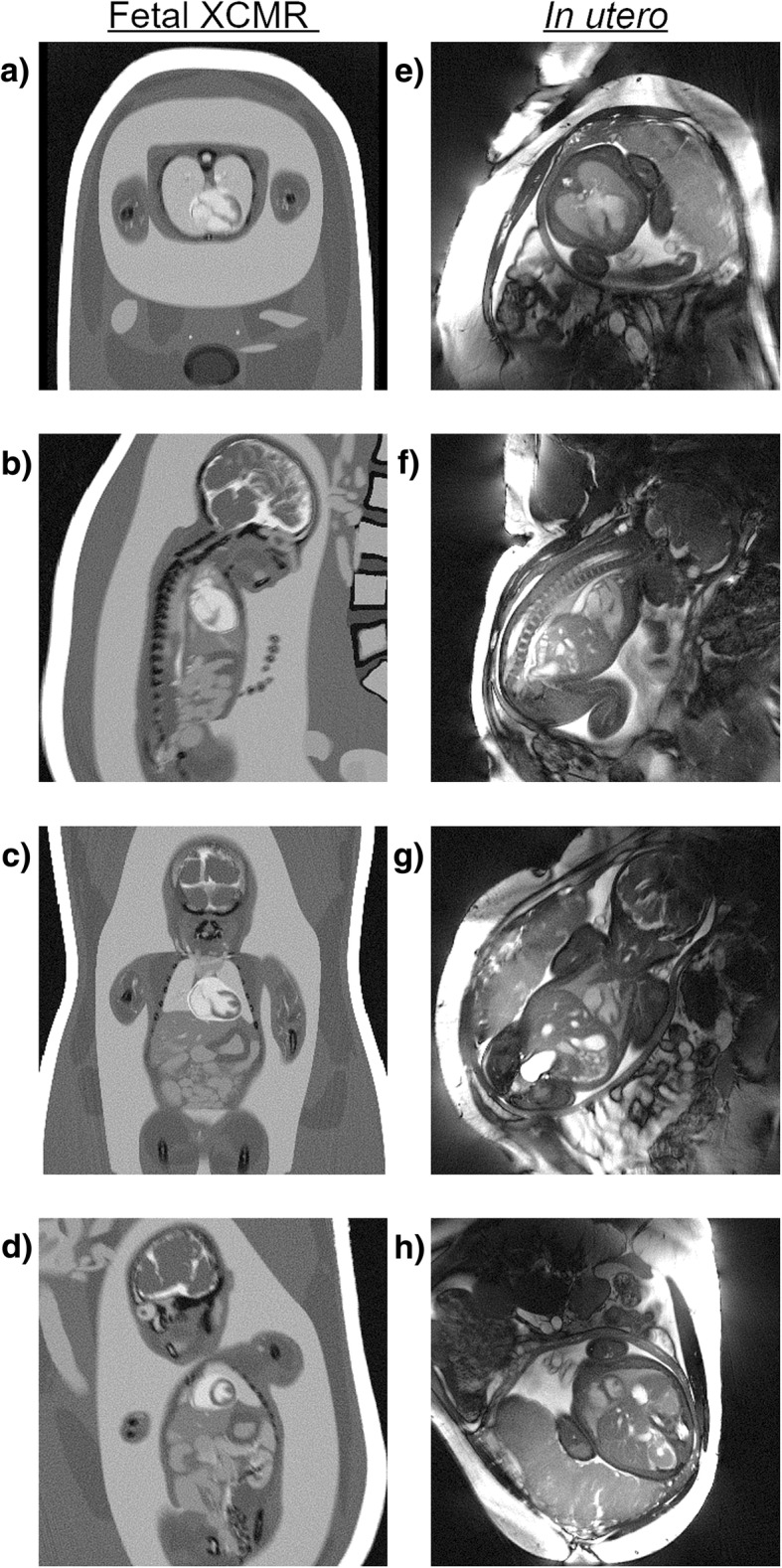
Static image reconstructions of Fetal XCMR (**a**-**d**) and in utero fetal (**e**-**h**) data demonstrating comparable maternal and fetal anatomy including fluid-filled lungs, amniotic fluid, and similar tissue signals and contrast. Reconstructions using all acquired spokes are shown for transverse (**a**, **e**), sagittal (**b**, **f**), coronal (**c**, **g**), and short-axis (**d**, **h**) orientations. In utero acquisitions were performed free breathing and Fetal XCMR motion was simulated accordingly. Consequently, these static images contain relatively blurred anatomy

Figure 3 displays representative static image reconstructions using the total number of acquired spokes from Fetal XCMR phantom and in utero fetal data sets. Overall, the morphologies and relative proportions of the maternal and fetal anatomy are well represented by the Fetal XCMR images in transverse (Fig. 3a), sagittal (Fig. 3b), coronal (Fig. 3c), and short-axis (Fig. 3d) orientations when compared to their in utero fetal image counterparts (Fig. 3e-h). Similarly, the CMR contrast is comparable between the two data types. Note that the fetal heart is relatively blurred in both simulate d and real data due to the underlying motion that is not resolved by the static reconstructions.

**Fig. 4:**
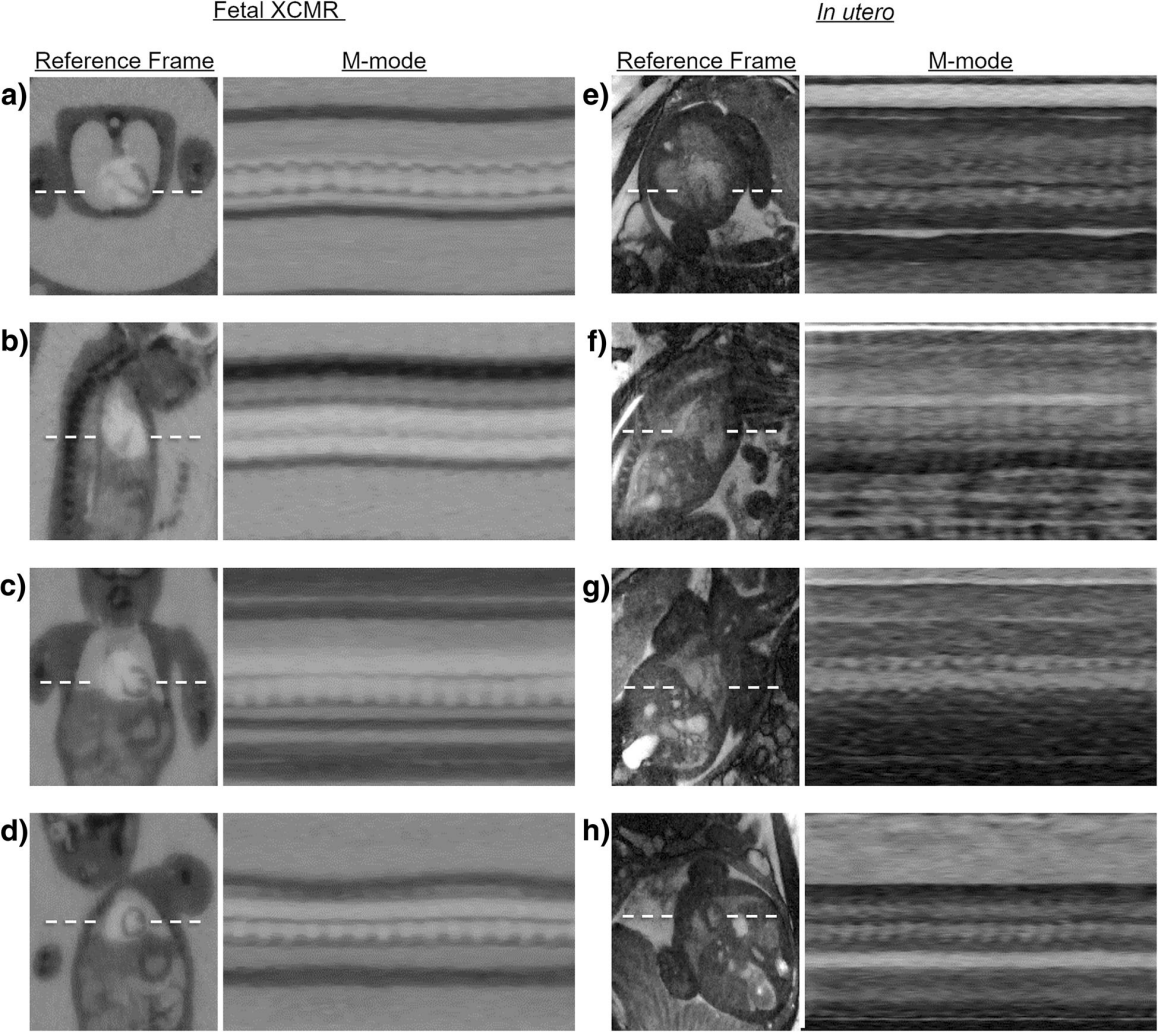
Real time image reconstructions of Fetal XCMR (**a**-**d**) and in utero fetal (**e**-**h**) data demonstrating comparable maternal and fetal anatomy and physiology. Movies corresponding to these reconstructions are available as supporting video S1. Reconstructions using a sequential sliding window are shown for transverse (**a**, **e**), sagittal (**b**, **f**), coronal (**c**, **g**), and short-axis (**d**, **h**) orientations. For each orientation and data type, a static frame is shown for reference along with an M-mode representation of the temporal dynamics along the dashed line


Figure 4 shows dynamic real-time image reconstructions of the same Fetal XCMR (Fig. 4a-d) and in utero fetal data (Fig. 4e-h) sets from Fig. 3. Movies corresponding to these reconstructions are available as Additional file 2: Video S2. For each data set, a still frame from the real-time image series is shown along with an M-mode representation of the temporal dynamics along the dash line. While the image quality is reduced, due to the limited number of spokes per image frame, the compressed sensing reconstructions allow for visualization of maternal respiratory and fetal cardiac motion. Note that negligible gross fetal movement was observed in the real fetal data sets and the related motion amplitudes for the Fetal XCMR simulation were set accordingly. Overall, the physiological motion is comparable between the simulated and real data sets.

Additional file 2:**Video S2.** Real time image reconstructions of Fetal XCMR (a-d) and in utero fetal (e-h) data demonstrating comparable maternal and fetal anatomy and physiology. Reconstructions using a sequential sliding window are shown for transverse (a, e), sagittal (b, f), coronal (c, g), and short-axis (d, h) orientations. (MP4 21600 kb)


**Fig. 5:**
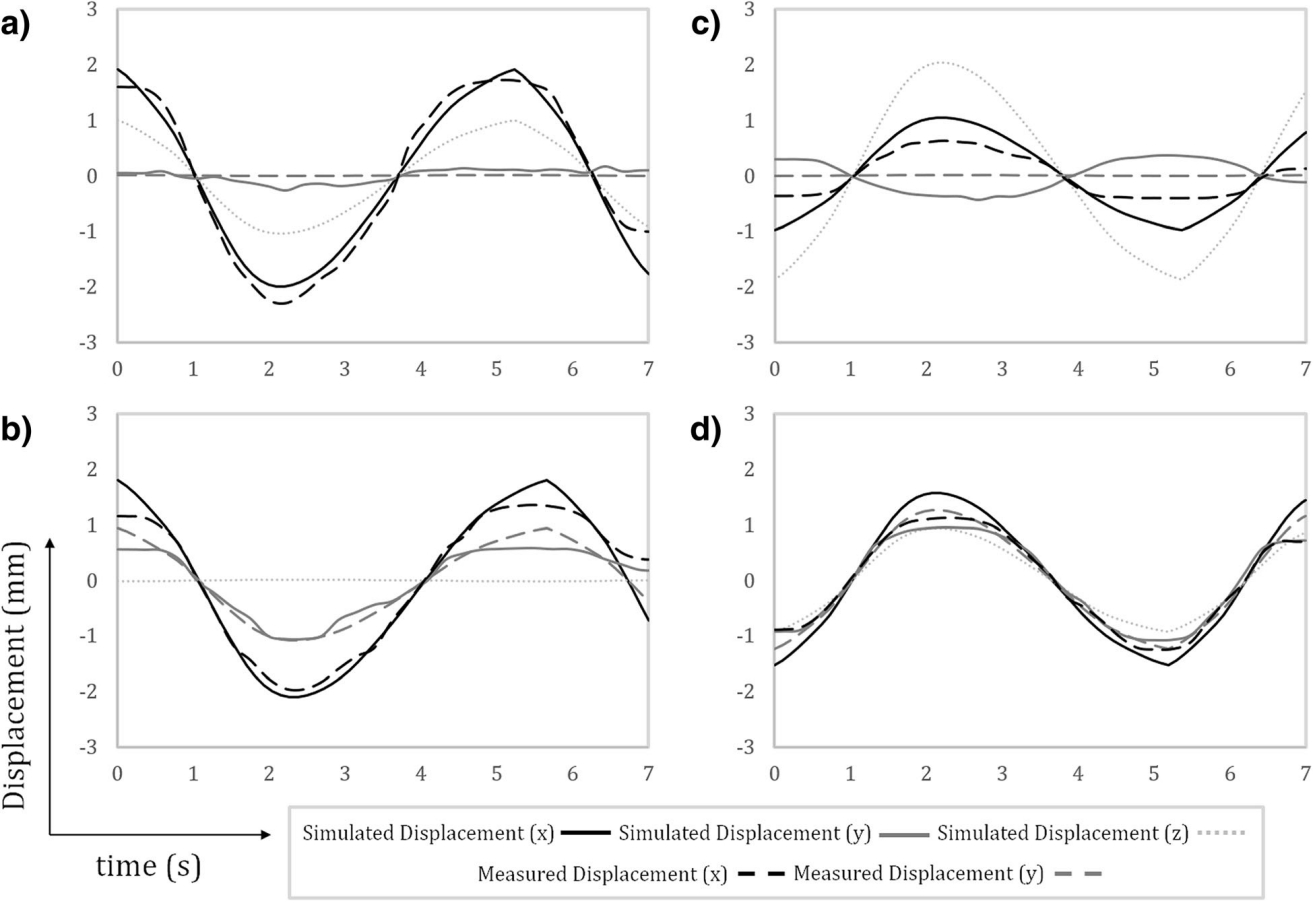
Motion estimates derived from translational registration of real-time image reconstructions of Fetal XCMR data in **a** transverse, **b** sagittal, **c** coronal, and **d** short-axis orientations. The ground truth simulated displacements are shown for all three spatial dimensions with corresponding measurements of in-plane displacement

For each real-time reconstruction of Fetal XCMR and in utero fetal data sets, in-plane displacement was estimated using translational registration of each frame of the real-time image series to a reference [3, 4]. Figure 5 plots the measured displacements from the four Fetal XCMR data sets as well as the ground truth displacements which are known from the simulation framework. In each case, the ground truth through-plane displacement is also shown but cannot be measured by the registration routine. Overall there is good agreement between the measured in-plane displacement and the simulated motion. However, only the sagittal slice orientation is unaffected by through-plane motion (there is no simulated respiratory motion along the x-axis of the 4D XCAT models) and therefore shows the best agreement. Note that this method for motion estimation has been previously demonstrated in fetal subjects and further validation using Fetal XCMR is beyond the scope of this paper. As such, Fig. 5 serves to illustrate that testing of image-based motion estimation is another potential application of the Fetal XCMR phantom.

**Fig. 6:**
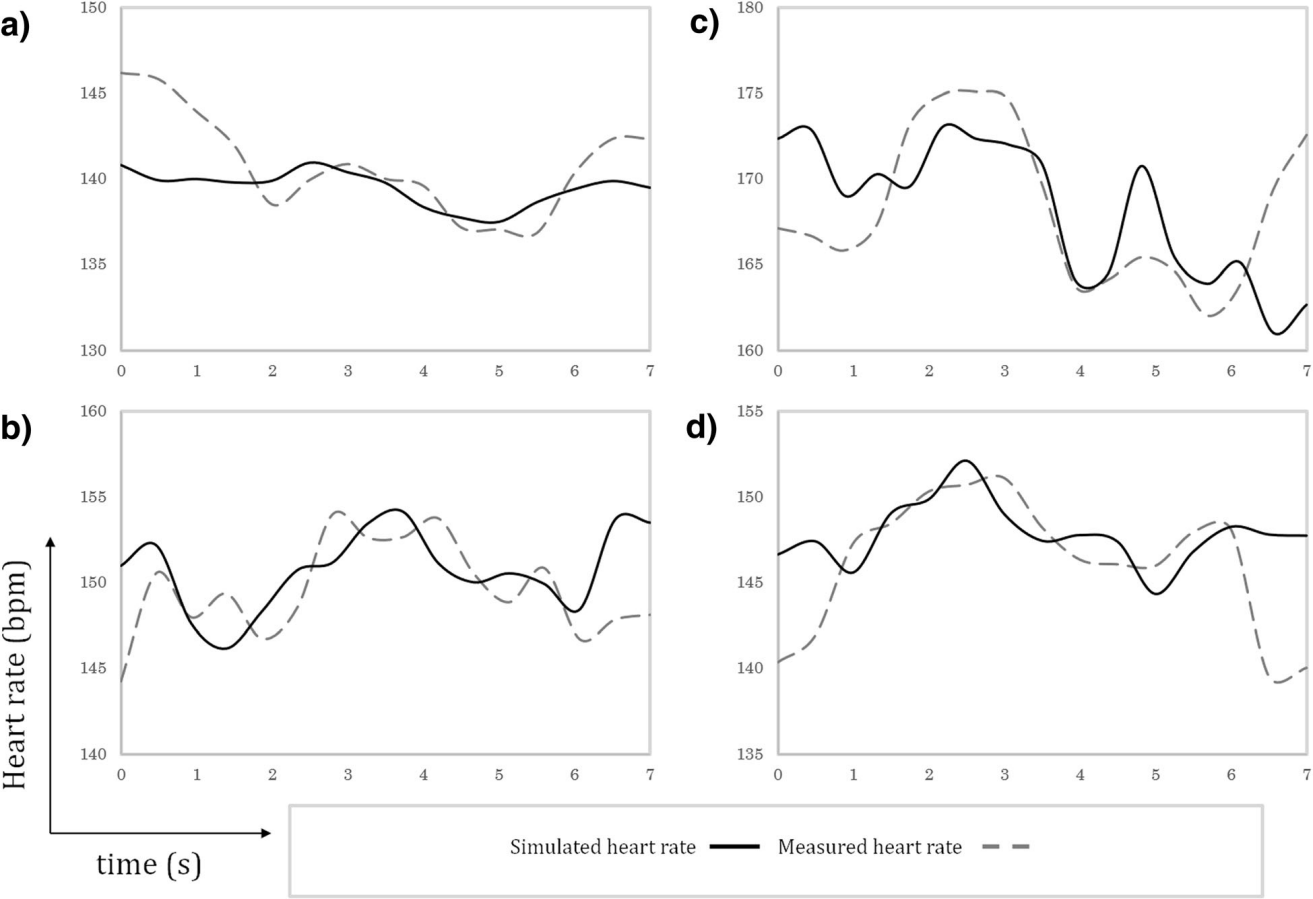
Heart rate estimates derived from real-time image reconstructions of Fetal XCMR data in transverse (**a**), sagittal (**b**), coronal (**c**), and short-axis (**d**) orientations. The ground truth simulated heart rates are shown along with the corresponding calculations obtained using metric optimized gating

In addition to measurement of in-plane displacement, real-time image reconstructions of Fetal XCMR and in utero fetal data sets were used to estimate the fetal heart rate using metric optimized gating [1, 3]. Figure 6 plots the measured heart-rates and ground truth simulation values for each simulated data set. Good agreement is shown between the measured and ground truth heart rates and the metric optimized gating algorithm can resolve the general trend in the heart rate variation over time.

As with the motion estimates shown above, metric optimized gating has been well established in fetal subjects and therefor Fig. 6 serves to illustrate that testing new image-based gating methods may be another potential application of the Fetal XCMR phantom.

**Fig. 7:**
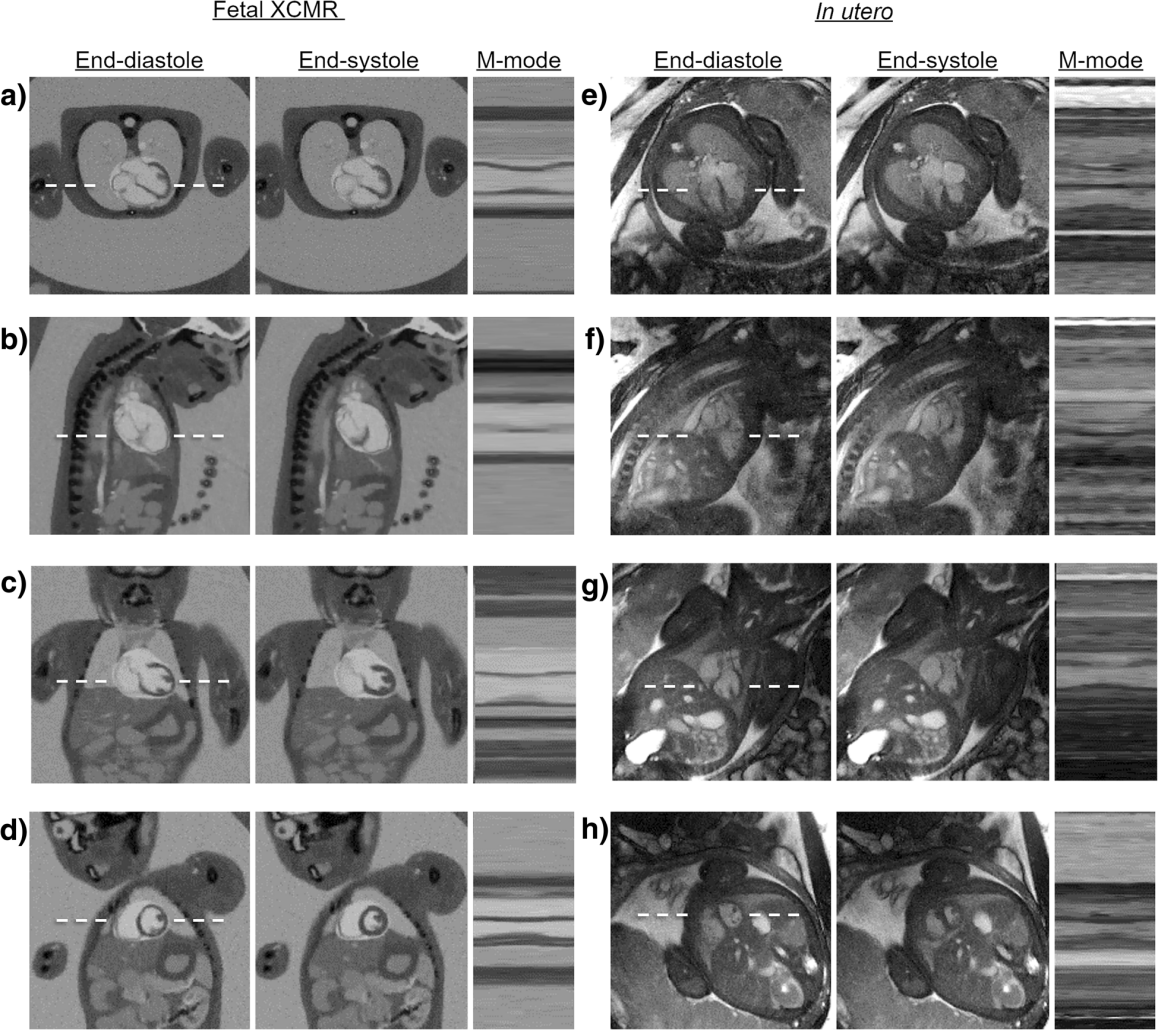
Motion Compensated CINE Reconstructions of Fetal XCMR (**a**-**d**) and In Utero fetal (**e**-**h**) data demonstrating high quality images of simulated and real fetal hearts. Movies corresponding to these reconstructions are available as supporting video S2. End-diastolic and end-systolic frames are shown in transverse (**a**, **e**), sagittal (**b**, **f**), coronal (**c**, **g**), and short-axis (**d**, **h**) orientations. For each orientation and data type, an M-mode representation of the temporal dynamics along the dashed line is also shown demonstrating fetal cardiac dynamics


Finally, the motion and heart rate estimates derived from the real-time images (Fig. 4) were applied to each of the corresponding simulated and real data sets to produce motion corrected CINE images of the fetal heart. Figure 7 shows high quality CINE image reconstructions of Fetal XCMR data sets in transverse (Fig. 7a), sagittal (Fig. 7b), coronal (Fig. 7c), and short-axis (Fig. 7d) orientations with corresponding in utero images shown for comparison (Fig. 7e-h). For each data set, end-diastolic and end-systolic frames from the CINE image series are shown along with an M-mode representation of the temporal dynamics along the dash line. Movies corresponding to these reconstructions are available as Additional file 3: Video S3. Overall, the CINE images provide excellent delineation of fetal cardiac anatomy and temporal dynamics for both data types. The cardiac dynamics are much better resolved in these CINE images relative to the real-time image reconstructions of the same data shown in Figs. 3 and 4, once again highlighting the potential application of the proposed simulation framework for comparing reconstruction methods.

Additional file 3:**Video S3.** Motion Compensated CINE Reconstructions of Fetal XCMR (a-d) and in utero fetal (e-h) data showing high quality images of simulated and real fetal hearts. Reconstructions are shown for transverse (a, e), sagittal (b, f), coronal (c, g), and short-axis (d, h) orientations demonstrating fetal cardiac dynamics. (MP4 11800 kb)


## Discussion

In this work, an open-source framework for simulating CMR images of the fetal heart was developed: Fetal XCMR. The Fetal XCMR phantom is based off the well established XCAT phantom and its extension to CMR imaging, MRXCAT [14, 24]. However, here we present a numerical phantom that captures the relative sizes and dynamic physiology of the fetal heart and fetal body in simulated in utero environment. User-selected parameters control standard CMR acquisition parameters as well as the level of maternal respiratory motion and gross fetal movement.

Several comparisons between the Fetal XCMR phantom and acquisitions of the fetal heart obtained in utero were made across four standard slice orientations (transverse, sagittal, coronal, short-axis) and three reconstruction methods (static, real-time, and motion-corrected CINE). In general, reconstructions of the Fetal XCMR phantom demonstrated realistic anatomy, motion levels, and image quality compared to the images of the fetal heart obtained in utero. Advanced image reconstructions methods that have been previously applied to fetal CMR, including compressed sensing, motion estimation, and image-based gating, were implemented for both the simulated and real data sets. While extensive validation of these methods is beyond the scope of this work, they serve to further demonstrate the potential use of the proposed framework. Nevertheless, improvements to the current phantom are discussed below.

The Fetal XCMR phantom is derived from two independent 4D XCAT tissue models of a maternal abdomen over a representative respiratory cycle and complete fetal anatomy over a representative cardiac cycle. These two tissue models are then interpolated to a given simulated acquisition time point, spatially shifted according to the underlying respiratory and gross fetal movement models and combined to form the base image for the simulated CMR acquisition. While the current framework provides a reasonable representation of maternal-fetal anatomy and motion, the current tissue models lack certain anatomical features. For example, there is no representation of the placenta, and the simulated fetal heart does not contain the blood vessels and shunts that are specific to the pre-natal circulation. Consequently, the fetal right ventricle is not accurately portrayed. For potential reconstruction applications of this phantom (i.e. improved image reconstruction or image-based gating), the relative size and dynamics of the Fetal XCMR anatomy should suffice. However, simulation of fetal cardiac pathologies which are complex and highly variable would require additional modifications.

The motion models used to simulate maternal respiration and gross fetal movement operate in all three spatial dimensions but are strictly translational. As a result, common fetal movements such as the bending of arms and legs or rotation of the torso are not represented in the current framework. Non-rigid motion is still simulated as through-plane motion in the 2D examples shown in the work; however, for extension to 3D imaging or for more accurate representations of fetal motion, XCAT tissue models could be modified to include variable fetal limb extension and compression albeit at an additional computational cost to generate additional 3D XCAT images for maternal respiratory, fetal cardiac, and non-rigid motion states.

The base spatial resolution of the current framework, which is defined by the resolution of the generated maternal and fetal XCAT tissue models, was set to 1 mm isotropic. Similarly, the base temporal resolution, which was defined by the number of uniquely generated timepoints, was set to 50 frames representing approximately 100 ms and 10 ms for representative maternal respiratory and fetal cardiac cycles respectively, depending on the simulated rates. Both the base spatial and temporal resolution used in this work reflect the lowest values currently reported in the literature for fetal CMR applications. Still, the base resolutions could be arbitrarily improved up to the native resolution of the XCAT tissue models of 0.3 mm isotropic, although such resolutions will come at a significantly increased memory burden.

The computational time required to generate individual Fetal XCMR data sets is relatively low (~ 10 min per slice using the scan parameters described for the comparison to in utero data). While the time will increase for additional numbers of coils, radial spokes or Cartesian frames, multi-slice acquisitions benefit from the fact that the maternal and fetal 4D XCAT images only need to be loaded once for a given orientation. Similarly, generating new 4D XCAT images (i.e. at a spatial resolution greater than the current implementation of 1 mm^3^) can be performed once, and saved for subsequent generation of Fetal XCMR data (80 + 30 min for the two tissue models). Nevertheless, for a simulation with many varied parameters, translating the framework from MATLAB to a more efficient coding language may be needed to decrease the computational burden.

The signal model implemented in the current simulation framework is for a bSSFP sequence. The extension to other sequences should be relatively straightforward to implement as the independent maternal and fetal 4D XCAT images can in principle be converted to CMR contrast with any user-defined signal equation. Additionally, the tissue relaxation properties were calculated for a 1.5 T field strength using values compiled from literature. Unfortunately, there are few reference standards for fetal relaxation properties and as such, the current framework could be improved with more accurate values as well as those at other field strengths (i.e. 3.0 Tesla).

 Although signal-to-noise (SNR) and coil geometry are user-modifiable in the current implementation, several hardware and physics factors affecting the signal and noise are not modeled by Fetal XCMR including field inhomogeneities, off-resonance artifacts, in-flow effects, slice profiles, and eddy-currents. However, the previously published MRXCAT framework employs a similar signal equation and has been well established as a useful method for evaluating CMR reconstruction techniques. While beyond the scope of this work, a more complex simulation of the CMR signal could be integrated into future versions of the Fetal XCMR framework. However, its inclusion should be coupled with a more extensive description of the maternal-fetal anatomy as the placenta has a significant effect on the magnetic field properties and consequently the SNR of the region containing the fetal heart.

Finally, the current simulation framework implements 2D Cartesian and radial trajectories, both of which have been used in previously reported fetal CMR applications. Furthermore, the modular structure of the Fetal XCMR framework facilitates future simulations using alternative, arbitrary trajectories (e.g. spiral, 3D, flow sensitive) and acceleration methods (e.g. parallel imaging, simultaneous multi-slice). As the field of fetal CMR continues to grow, new methods will become available and require careful validation. The Fetal XCMR phantom is therefore a powerful and convenient tool in the continued development of fetal cardiac imaging.

## Conclusions

The Fetal XCMR phantom provides a new method for evaluating fetal CMR acquisition and reconstruction methods by simulating the underlying anatomy and physiology. Applications to static and dynamic imaging as well as image-based motion estimation and retrospective gating have shown potential applications of the phantom. Given the modular nature of the proposed framework, extensions to other trajectories and imaging techniques is possible, enabling the development of new methods for assessing the fetal heart in utero.

## Abbreviations

2D: Two-dimensional
4D: Four-dimensional
bSSFP: Balanced steady state free precession
CMR: Cardiovascular magnetic resonance
Fetal XCMR: Fetal extended Cardiac-Torso cardiovascular magnetic resonance
NUFFT: Non-uniform fast
SNR: Signal-to-noise ratio
XCAT: Extended Cardiac-Torso
